# Hyperkalemia-Induced T Wave Oversensing in an Implantable Cardioverter Defibrillator

**DOI:** 10.7759/cureus.54135

**Published:** 2024-02-13

**Authors:** Ariel Gonzalez, Hilton Franqui, Jose Lopez, Hector Banchs

**Affiliations:** 1 Medicine, University of Puerto Rico, Medical Sciences Campus, San Juan, PRI

**Keywords:** t wave oversensing, cardiac electrophysiology, implantable cardioverter-defibrillator (icd), cardiac implantable devices, severe hyperkalemia

## Abstract

A 66-year-old female with end-stage renal disease and heart failure with reduced ejection fraction, status post implantable cardioverter defibrillator (ICD) presented to the emergency department with dizziness and fatigue. An electrocardiogram showed sinus rhythm, complete atrioventricular block, and ventricular paced rhythm at 30 beats per minute (bpm). Device interrogation revealed a programmed VVI mode with a lower rate limit of 40 bpm and evidence of T wave oversensing. Serologic studies were remarkable for hyperkalemia (7.9 mmol/dL). The device was initially reprogrammed to provide a higher pacing rate and symptomatic improvement. Both complete AV block and T wave oversensing resolved after correction of hyperkalemia. This case highlights the need for vigilant monitoring of electrolyte imbalances in ICD patients.

## Introduction

T wave oversensing has been reported to occur in up to 14% of patients, and it remains a major issue in contemporary implantable cardioverter defibrillators (ICDs) [1]. It may result in inappropriate shock delivery and inappropriate device pacing inhibition as demonstrated by the present case. Hyperkalemia is known to increase the amplitude of the T wave. We present a case in which hyperkalemia resulted in T wave oversensing, leading to ventricular pacing inhibition in the setting of a complete AV block.

## Case presentation

A 66-year-old Hispanic female with type 2 diabetes mellitus, systemic arterial hypertension, end-stage renal disease on hemodialysis, and heart failure with reduced ejection fraction (EF: 30-35%) presented to the emergency room due to dizziness of four hours’ evolution. The patient denied chest pain, neurological changes, or fever. The patient received her last dialysis 48 hours prior to the onset of symptoms. On physical evaluation, blood pressure was 80/40 mmHg and heart rate was 30 beats per minute (bpm). The patient had euvolemic state, clear lungs, and no jugular vein distention. Serum creatinine level was 4.3 mg/dL, blood urea nitrogen (BUN) was 70 mg/dL, and potassium level was 7.9 mmol/dL (Table 1).

**Table 1:** Relevant laboratory parameters at presentation.

Laboratory parameters	Value	Normal reference range
Serum creatinine	4.3 mg/dL	0.84-1.21 mg/dL (varies by age/gender)
Blood urea nitrogen (BUN)	70 mg/dL	7-20 mg/dL
Potassium level	7.9 mmol/dL	3.5-5.0 mmol/dL

**Figure 1:**
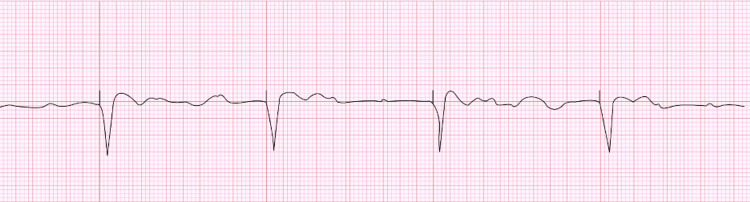
Ventricular paced rhythm at 30 bpm with sinus rhythm at 80 bpm with evidence of complete atrioventricular block. Mode: VVI, sensitivity at 0.3 mV, and lower rate limit of 40 bpm.

**Figure 2:**
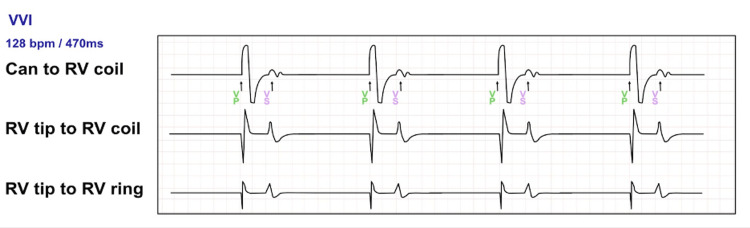
Interrogation of ICD with evidence of ventricular pacing with oversensing of the T wave while on RV sensitivity parameters of 0.30 mV and lower rate limit at 40 bpm. Mode: VVI. ICD: implantable cardioverter defibrillator; RV: right ventricle

The electrocardiogram revealed a complete atrioventricular block with ventricular-paced rhythm of 30 beats per minute (Figure 1). After failing to respond to a 1000 mL normal saline infusion, an intravenous dopamine infusion was started at 10 mcg/min. Oral kayexalate, albuterol, and intravenous calcium gluconate were administered, followed by urgent hemodialysis. Device interrogation demonstrated a VVI mode with a lower rate interval of 40 beats per minute (Figure 2).

**Figure 3:**
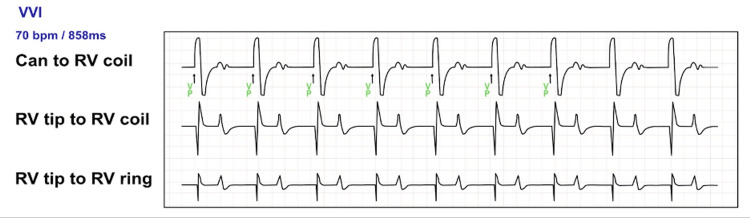
Interrogation of ICD with evidence of ventricular pacing at 70 bpm with appropriate R wave sensing after RV sensitivity was set to 1.2 mV and a lower rate limit of 70 bpm. Mode: VVI. ICD: implantable cardioverter defibrillator; RV: right ventricle

Electrogram tracings showed perceived T waves as ventricular activity at 128 bpm, as labeled in the marker channel. There was no evidence of T wave oversensing on previous tracings on the event log. While correcting hyperkalemia, sensitivity was increased from 0.3 mV to 1.2 mV and the lower rate limit was increased to 70 bpm (Figure 3). This resulted in consistent ventricular pacing at 70 bpm. AV block and T wave oversensing were resolved once the potassium level had normalized. The device was reprogrammed to 0.6 mV.

## Discussion

**Figure 4:**
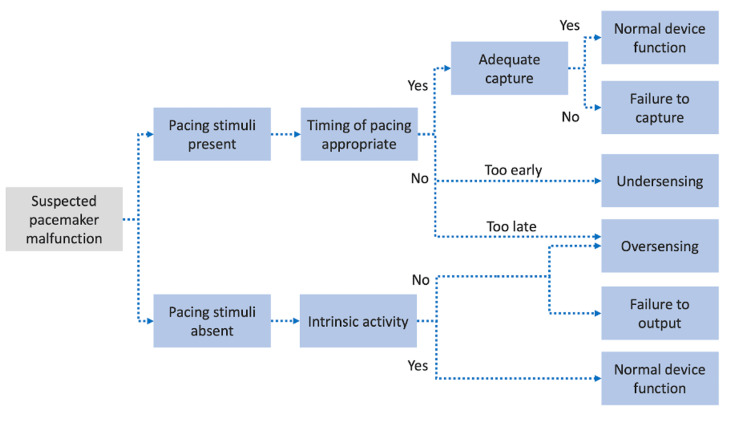
Approach to pacemaker function troubleshooting.

Hyperkalemia is an electrolyte disturbance that can cause tall, broad-based symmetrical T waves, inter-ventricular conduction delays or blocks, PR segment prolongation, and sinus arrest. These acute electrographic changes can cause cardiac implantable electronic devices (CIED) to respond inappropriately to intracardiac signals [2]. Figure 4 describes an algorithm for pacing function troubleshooting [3].

Patients may experience inappropriate shocks, unintended pacing inhibition, and extended effective pacing intervals, resulting in severe bradycardia that can become symptomatic in individuals who are pacemaker-dependent [4].

In the present case, the device was pacing at 30 bpm, despite a programmed lower rate limit of 40 bpm, due to T wave oversensing, as evidenced by device interrogation. Oversensing, particularly of the T wave, is more common in ICDs, as programmed sensitivity is usually higher and consists of a decreasing slope rather than a fixed value compared to pacemakers. T-wave oversensing is further increased if a patient has a baseline low amplitude R wave, prolonged QT interval, bundle branch blocks, or paced rhythm. There are reported cases where T wave oversensing can occur in hyperacute T waves, Brugada syndrome, diaphragmatic myopotential artifacts, and intraventricular conduction delay after alcohol ablation [5].

To lower the incidence of T wave oversensing, physicians can decrease sensitivity in ICDs, adjust post ventricular atrial refractory period, and/or implement dedicated to integrated bipolar systems [6]. In the present case, we decreased the ventricular sensitivity and caused an immediate increase in ventricular pacing to the programmed lower rate limit of 70 bpm. After emergent dialysis, the patient experienced complete resolution of the AV block and returned to appropriate T wave discrimination.

## Conclusions

Clinically symptomatic T wave oversensing is rare, but it may cause significant harm when present. This case highlights inappropriately low ventricular pacing in the setting of transient complete AV block secondary to T wave oversensing.
